# Supplementary material for: Electron cryo-microscopy reveals the structure of the archaeal thread filament
Source: Nat Commun. 2022 Dec 1;13:7411. doi: 10.1038/s41467-022-34652-4 (PMC9715654; doi:10.1038/s41467-022-34652-4)
Supplement: Supplementary file 1 — Supplementary Information [file 41467_2022_34652_MOESM1_ESM.pdf]

Supplementary figure 1

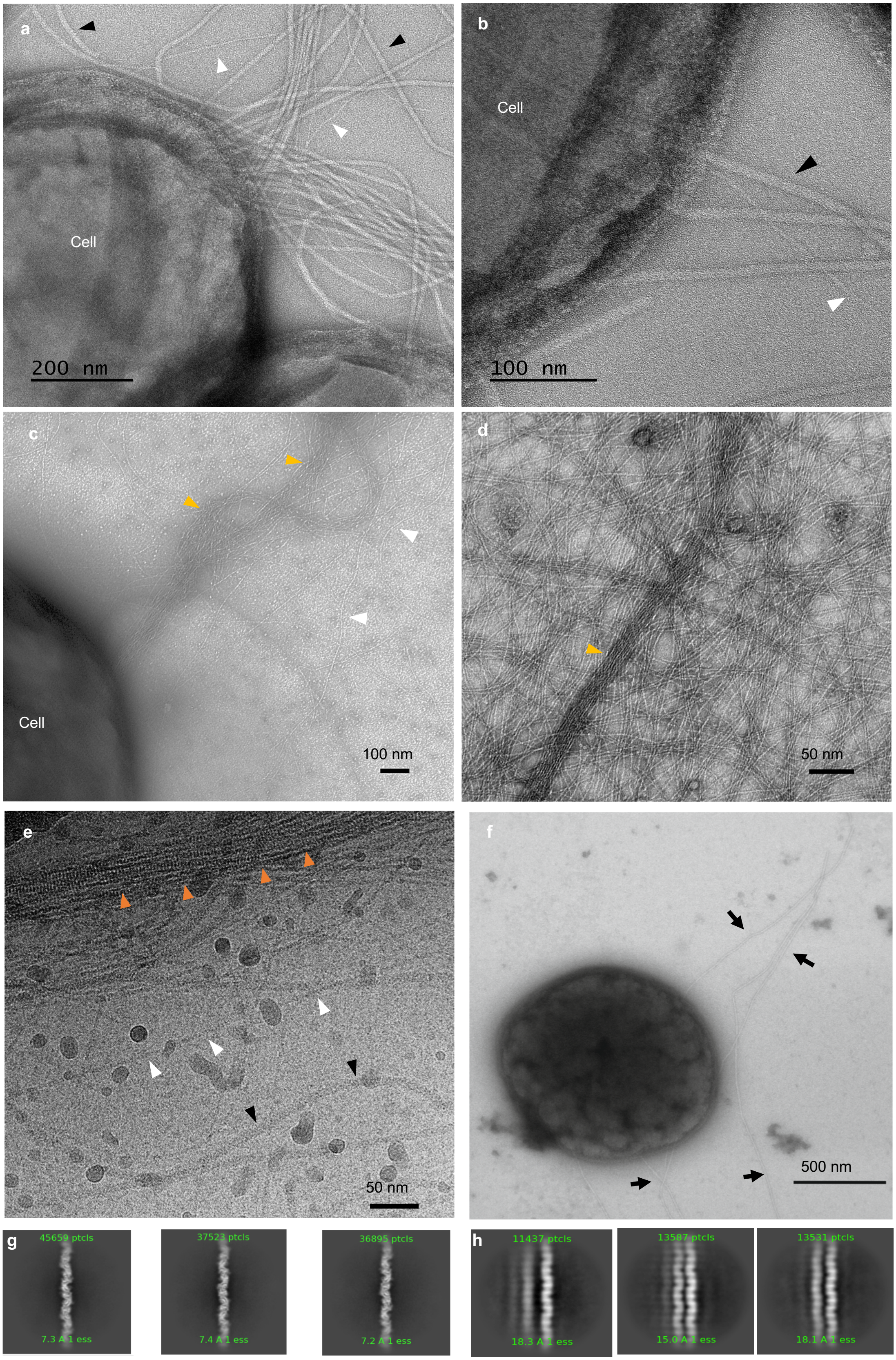

Supplementary Figure 1 – Negative Stain transmission electron microscopy of *S. acidocaldarius* MW001, ΔpibD and Δ*saci0405_saci0406* mutants.

a, b, micrographs of the cellular periphery of *S. acidocaldarius* MW001 cells at different magnifications. White arrowheads, threads; black arrowheads Aap or archaella. **c,** micrograph of Δ*pibD* mutant that lacks archaella and Aap but retains threads. Orange arrowhead indicates a cable of threads. **d,** micrograph of isolated thread filaments. Individual filaments and cables are evident. **e,** cryoEM micrograph showing a mixture of isolated *S. acidocaldarius* threads and AAP. White arrowheads, threads; orange arrowheads, cables of threads; black arrowheads, AAP or archaella. **f,** Δ*saci0405_saci0406* knockout mutant shows only AAP / archaella (black arrows) but no threads**. g,** 2D classification of thread filaments carried out in cryoSPARC. **h**, 2D classification showing the tendency of threads to line up in parallel cables. Data shown in these micrographs were reproducible across several (>3) grids for each sample. Micrographs were taken of cells and filaments from several (10-20) areas of the grids.

Supplementary figure 2

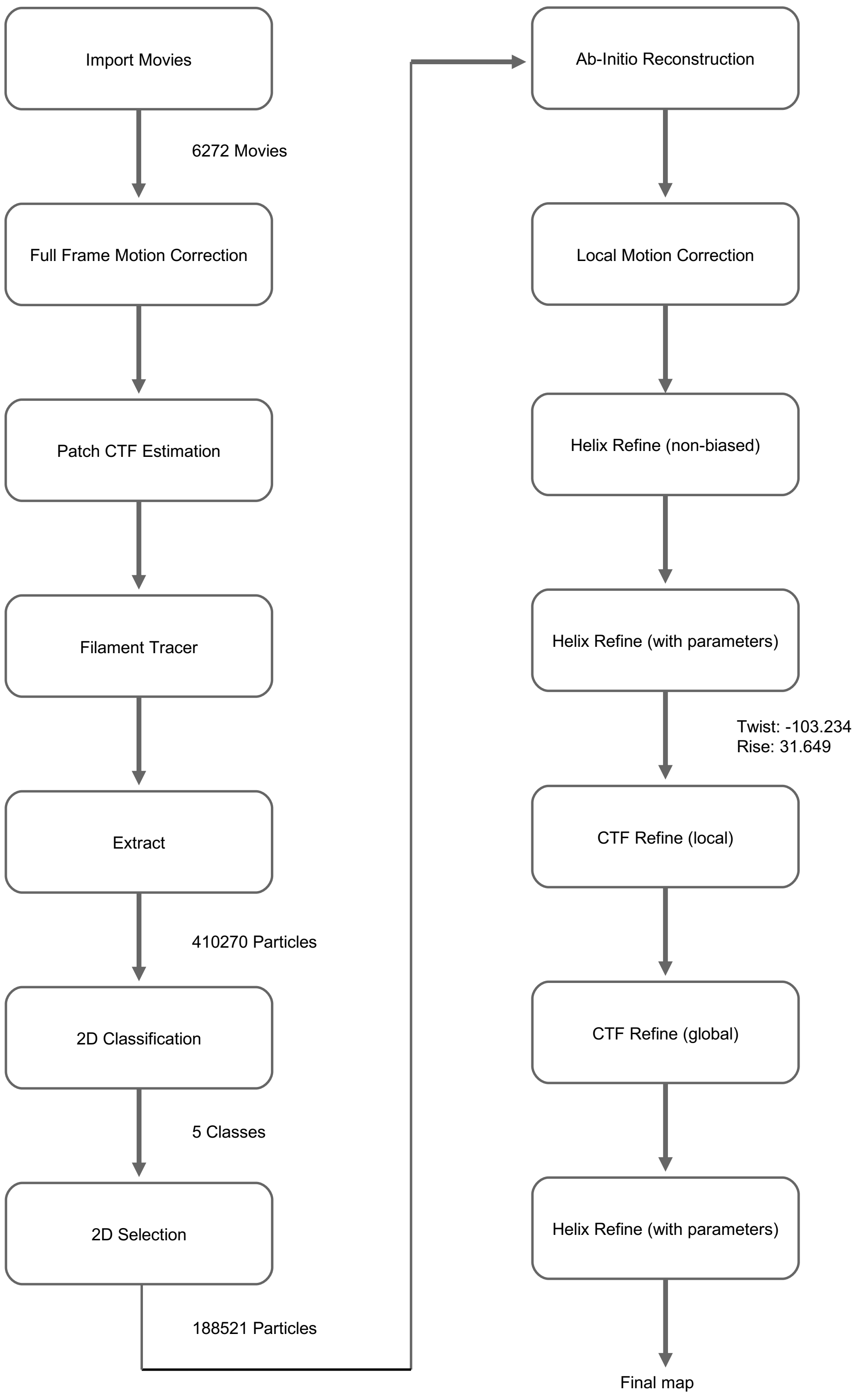

Supplementary Figure 2 – Image processing pipeline
Flowchart showing the helical reconstruction workflow in cryoSPARC[1]

Supplementary figure 3

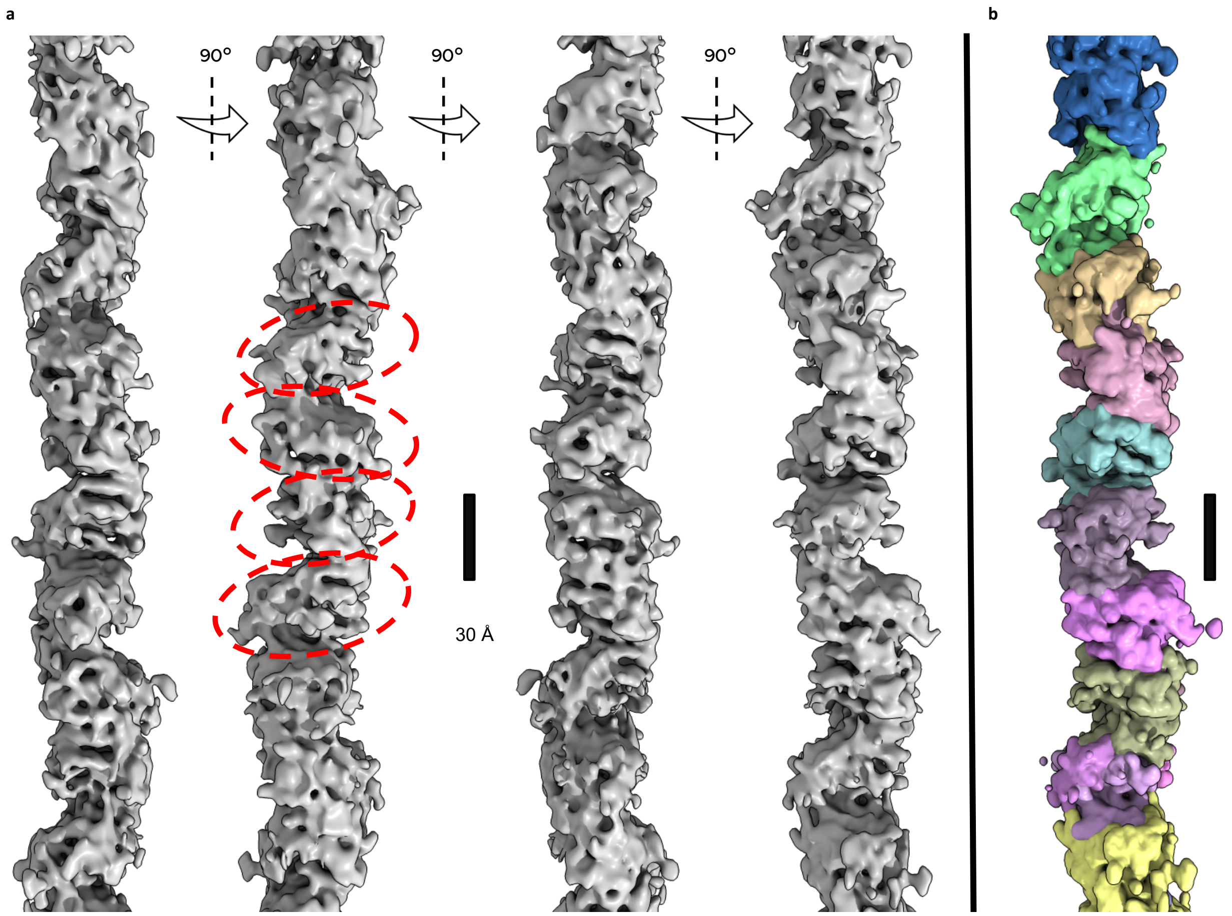

Supplementary Figure 3 – 3D reconstruction without helical parameters

a, Unbiased helix refinement generated in cryoSPARC[1] without the input of helical parameters. Careful inspection of the density indicates that the subunit rise is approximately 30 Å (black scale bar). Individual subunits are indicated with red, dashed ovals. **b**, segmented map showing the filament's subunits in various colours. Scale bar 30 Å

Supplementary figure 4

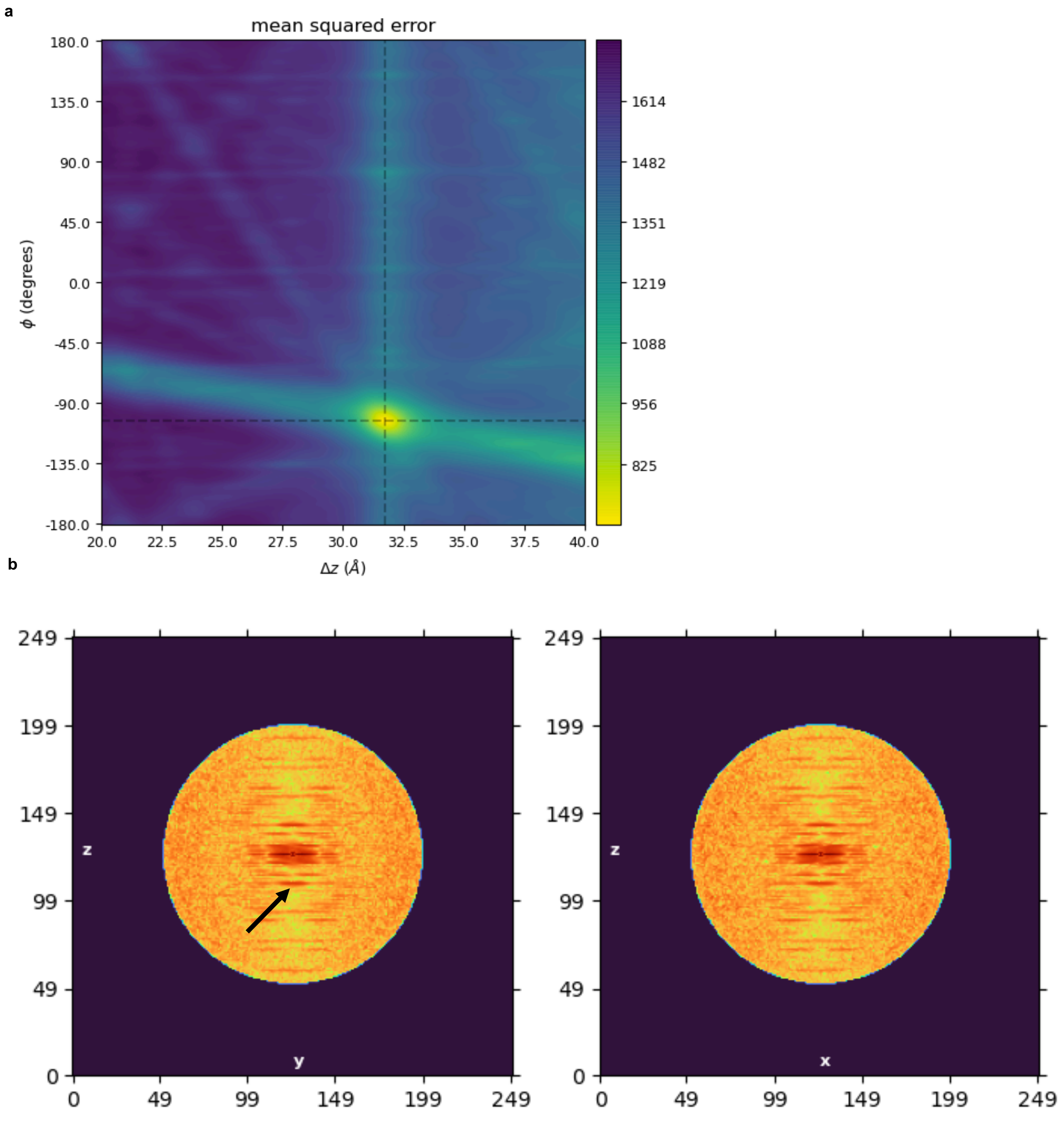

Supplementary Figure 4 – Helical parameter search

a, cryoSPARC[2] symmetry search showing a clear peak at 31.6 Å rise and -103° twist. **b**, the helical rise is confirmed by a meridional reflection (black arrow) in the power spectrum of the thread filament, which corresponds to a value of ~31.6 Å.

Supplementary figure 5

a

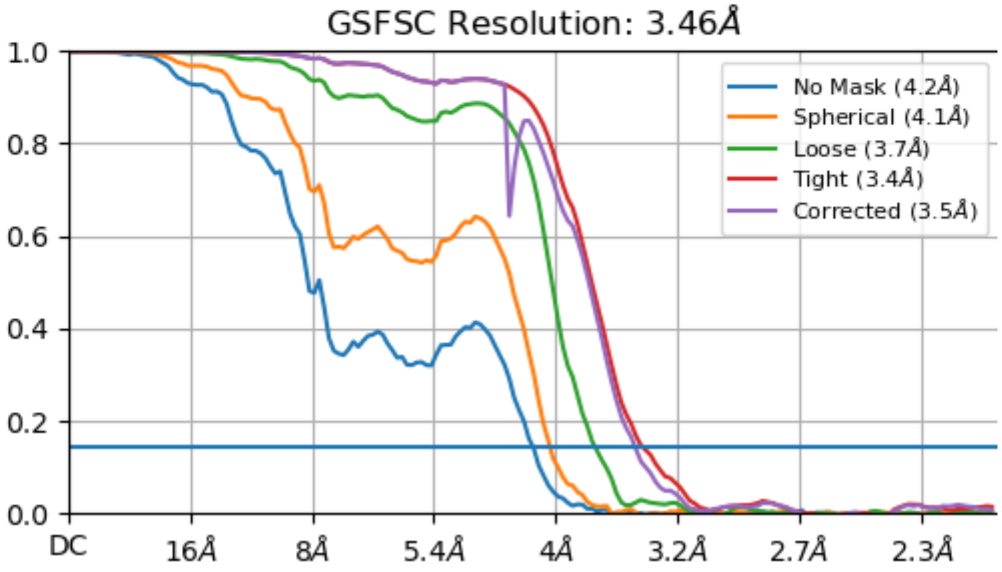

b

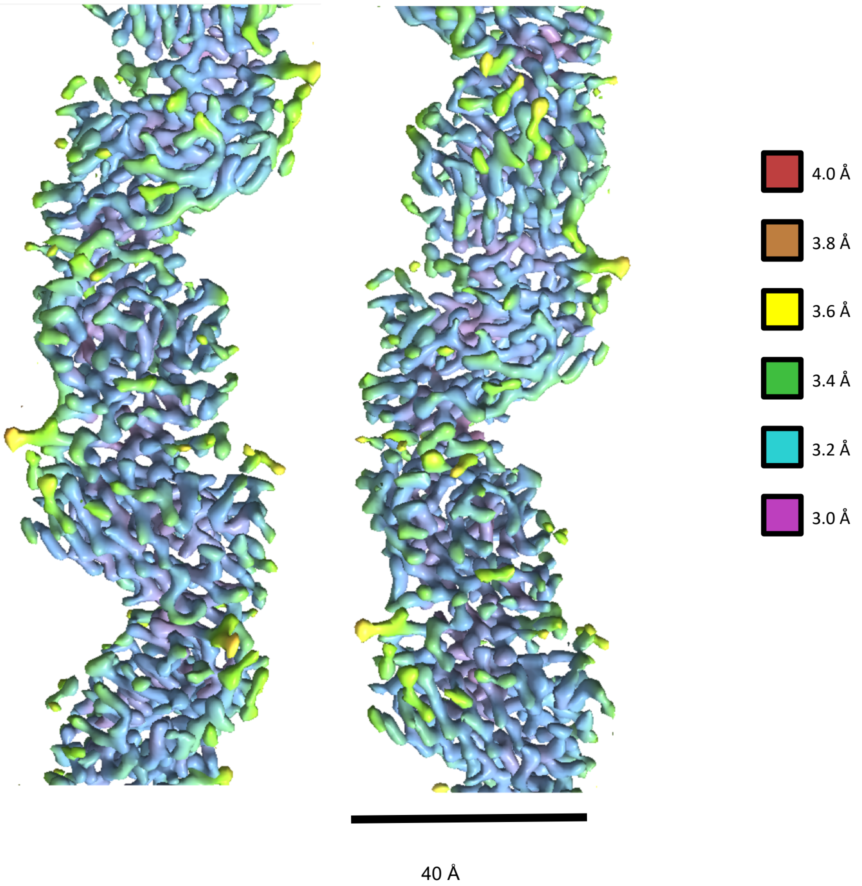

Supplementary Figure 5 – Global and local resolution estimation

a, global resolution of the helical reconstruction of the thread filament. **b,** local resolution map indicates a resolution range of ~3 – 4 Å. The core of the filament is resolved best, while peripheral regions are slightly less well defined. This is particularly the case for some of the flexible glycan moieties. Scale 40 Å

Supplementary figure 6

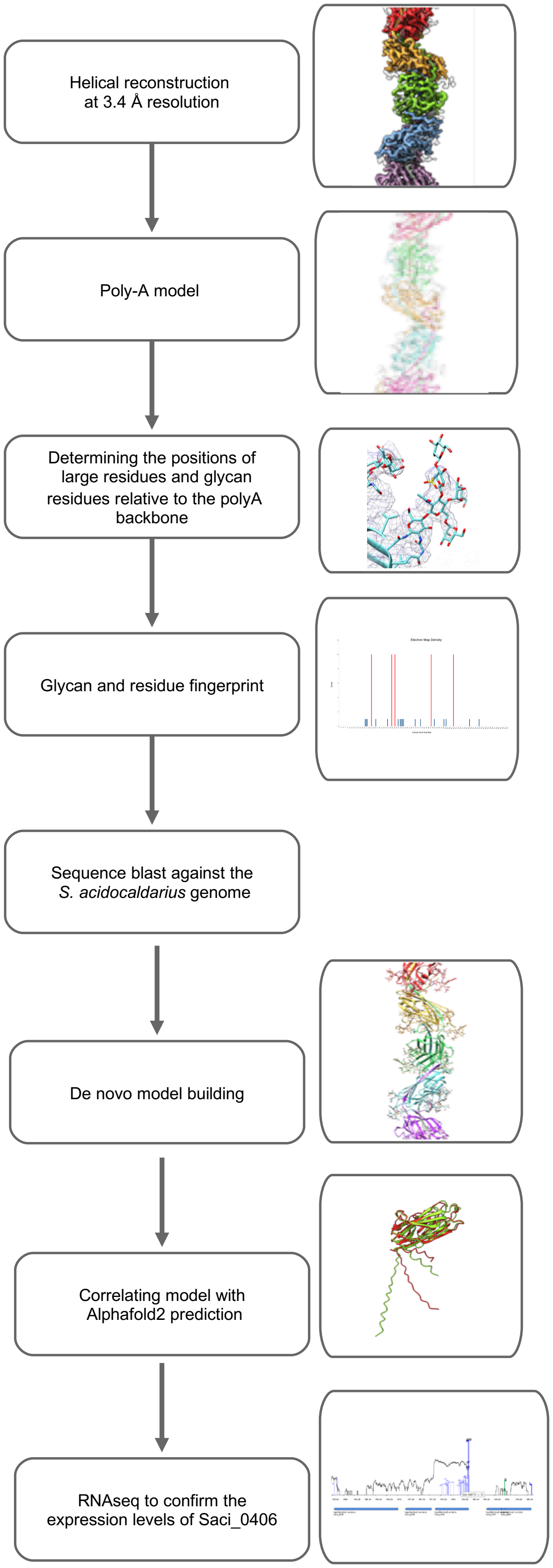

Supplementary Figure 6 – Determining the identity of the thread subunit protein from the cryoEM map. Flow chart illustrating how the identity of the thread subunit Saci_0406 was revealed based on the cryoEM map

Supplementary figure 7

a

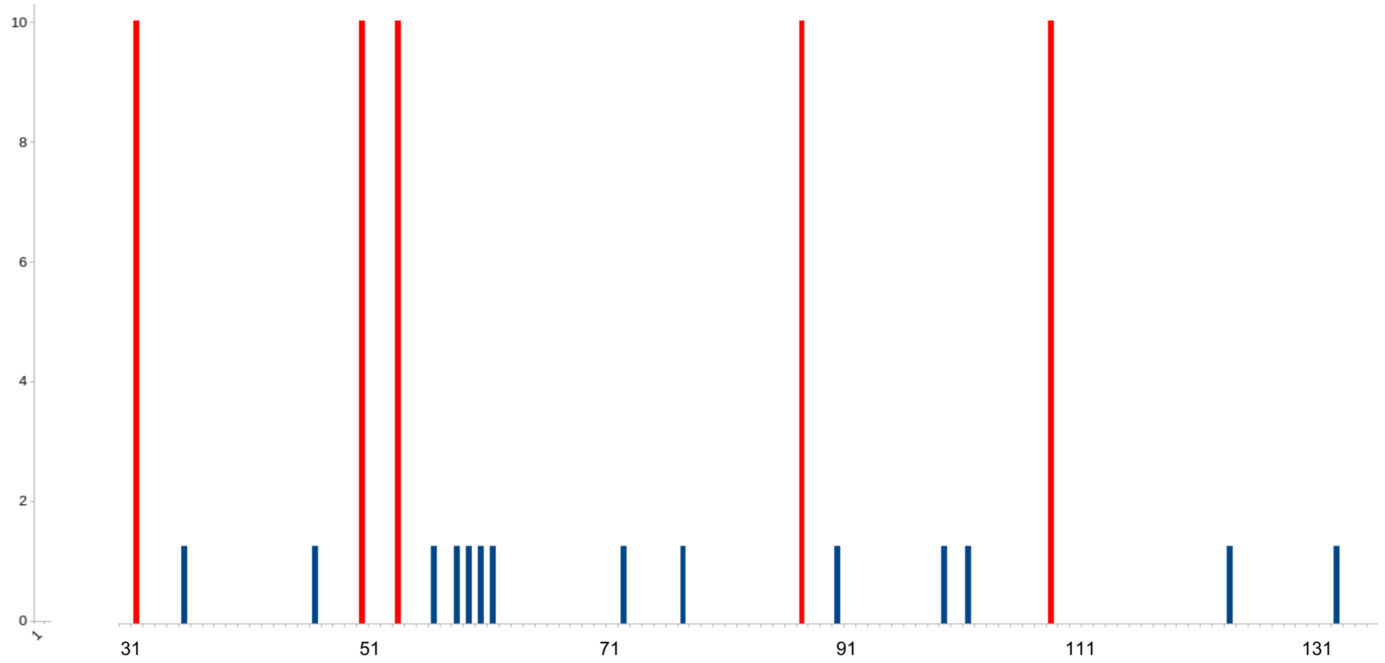

b

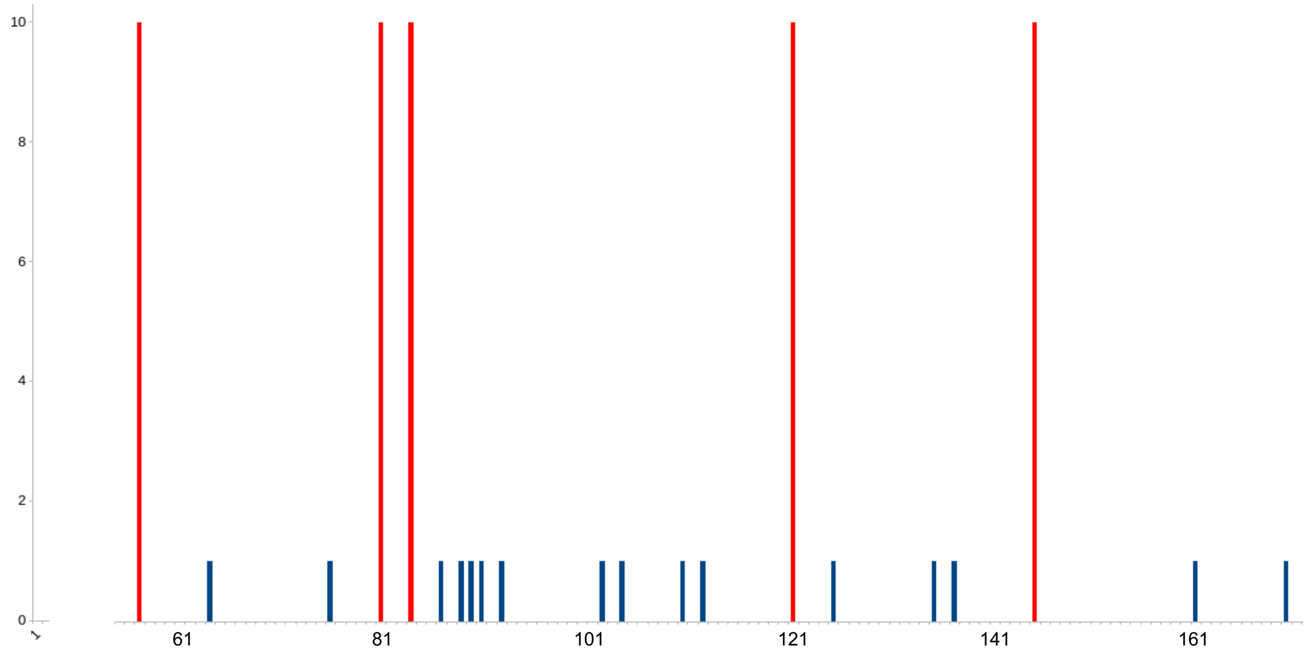

Supplementary Figure 7 – Glycan and large residue fingerprint

a, bar chart illustrating the positions of large side chains and putative glycan densities relative to a poly-alanine chain treaded though the cryoEM map. For illustration purposes, glycans were given a value of 10 (red bars) and visibly aromatic amino acid residues a value of 1 (blue bars). **b,** bar chart illustrating the position of conserved glycosylation sequons (**N**XS/T) (red) and aromatic residues (blue) within the sequence of Saci_0406. Note that the sequence in (**a**), is longer, as it contains the N-terminal signal sequence, which is cleaved in the mature protein.

Supplementary figure 8

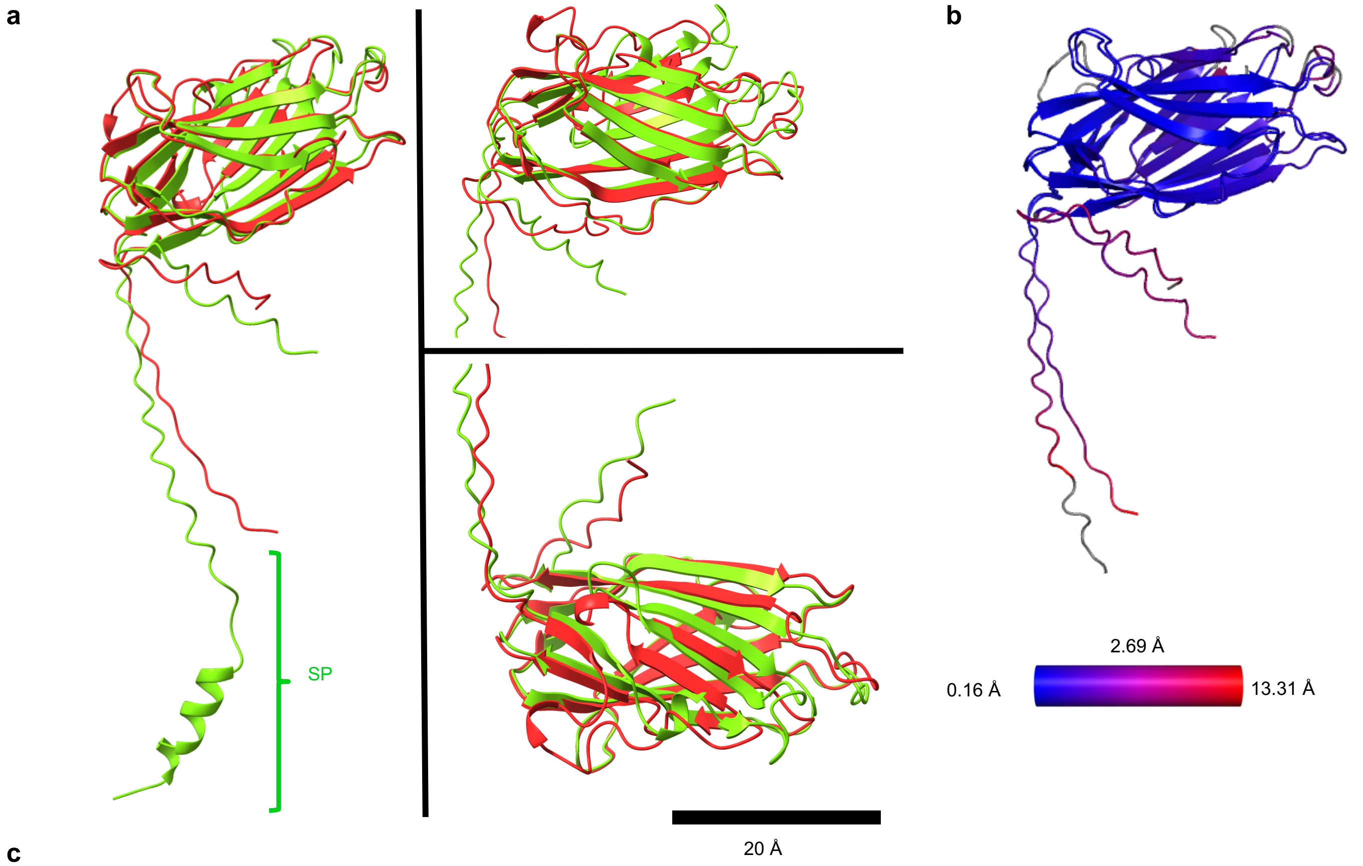

c

```
1   MNKKRLLLLS VFLVSVFVPV LVADVIYYYQ GQITVGNVAP PMYFAIQPNG NAKIGNNSNV
61  PSYINAQPSS GGSGFTAQVN ITNATYNYYF NFMGLAVSKT GYIYLAKVAY SYTATNNPIQ
121 NATLYIMNQQ GQIVYKYKLI VNGVVNSTLP STPLQINSGS YIVSLLIVPY QGTLPKTPSN
181 DLATITVNFG FSPMTASPPP IPLPSP
```

Supplementary Figure 8 – *Ab initio* structure vs Alphafold2 prediction of Saci_0406

a, *ab initio* atomic model of Saci_0408 (red) superimposed with the Alphafold2[3] prediction of the protein (green). Note that the Alphafold2 prediction additionally contains the N-terminal signal peptide (SP). **b**, RMSD values between the two structures in A (blue, low; red high RMSD). Average value 2.69 Å. **c**, Protein sequence of Saci_0406 with the cleaved signal sequence highlighted in yellow and glycosylation sites in green. Each of the glycosylated asparagine residues resides in a NXS/T sequon. Scale bar 20 Å

Supplementary figure 9

a

```
>Saci_0406
MNKKRLLLLSVFLVSVFVPVLVADVIYYYQGQITVGNVAPPMYFAIQPNGNAKIGNNSNV
PSYINAQPSSGGSGFTAQVNITNATYNYYFNFMGLAVSKTGYIYLAKVAYSYTATNNPIQ
NATLYIMNQQGQIVYKYKLIVNGVVNSTLPSTPLQINSGSYIVSLLIVPYQGTLPKTPSN
DLATITVNFGFSPMTASPPPIPLPSP
```

(Highlighted in magenta: **MNKKRLLLLSVFLVSVFVPVLVA**)

b

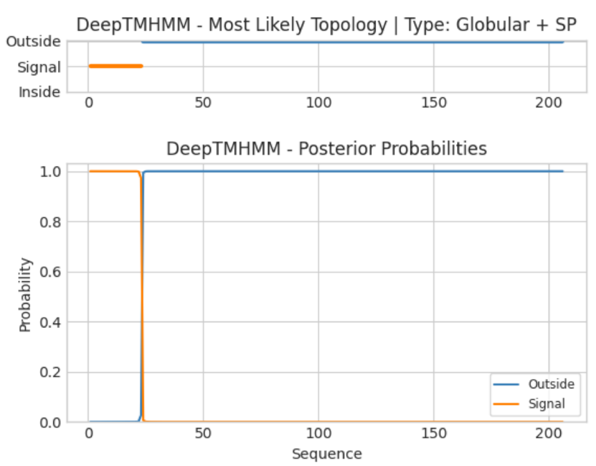

c

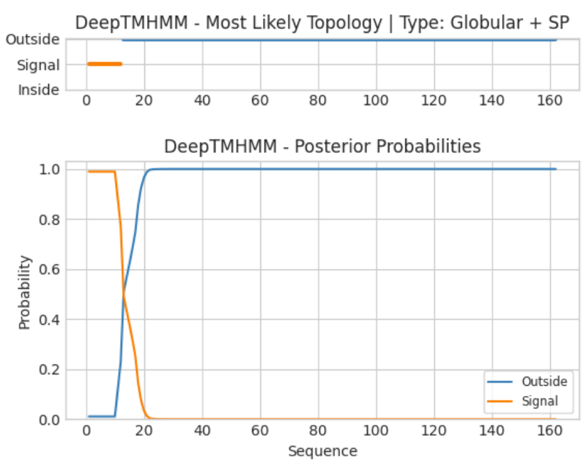

d

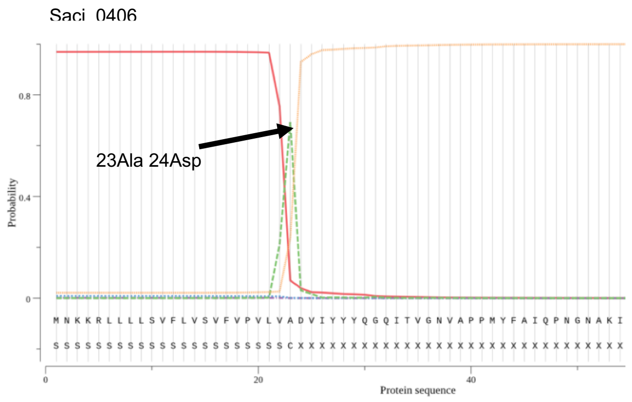

Protein Type	Signal peptide Sec/SPI<br><br>Red	TAT signal peptide Tat/SPI<br><br>Purple	Lipoprotein signal peptideSec/SPII<br><br>Blue	Other<br><br>Yellow
Likelihood	97%	0.1%	0.8%	2.1%

e

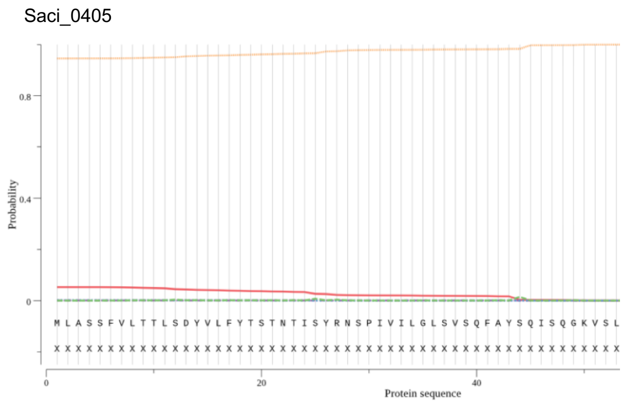

Protein Type	Signal peptide Sec/SPI<br><br>Red	TAT signal peptide Tat/SPI<br><br>Purple	Lipoprotein signal peptideSec/SPII<br><br>Blue	Other<br><br>Yellow
Likelihood	5.3%	0.08%	0.1%	94.52%

Supplementary Figure 9 – Signal sequence prediction using the SignalP 5.0 server

a, Sequence of Saci_0406 with the signal sequence highlighted in magenta. **b,** DeepTMHMM[4] prediction for Saci_0406 indicates the signal sequence highlighted in a. **c,** DeepTMHMM prediction for Saci_0405. **d,** SignalP-5.0 prediction indicates a SEC for Saci_0406, which is cleaved by signal peptidase 1 (SP1) **e**, SignalP-5.0 prediction for Saci_0405 shows no SEC signal sequence.

Supplementary figure 10

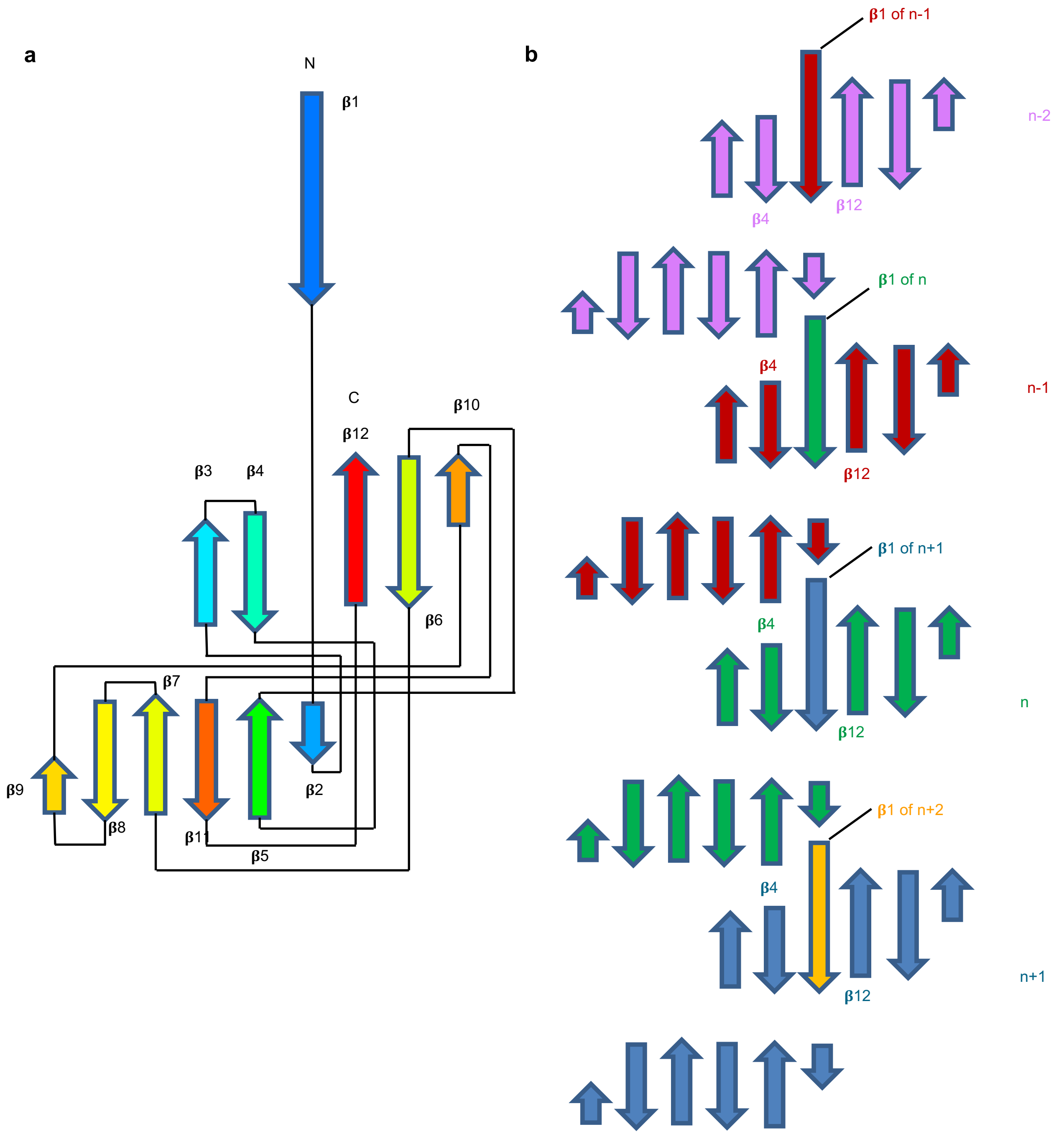

Supplementary Figure 10 – Topology diagram of the thread

a, topology of Saci_0406 in rainbow colouring (blue, N-terminus; red, C-terminus). The subunit consists of 12 **β** strands (numbered). A gap between **β**4 and **β**12 serves as **β**-strand acceptor site. **β**1 acts as **β**-strand donor for an adjacent monomer. **b**, topology of four Saci_0406 subunits within the thread. The central monomer is named n. n-1 is the monomer preceding n and n+1 is the monomer following n. The monomer n inserts its **β**-strand into the acceptor site between **β**4 and **β**12 of the monomer n-1. The monomer n+1 inserts its **β**-strand into the acceptor site between **β**4 and **β**12 in the monomer n and so forth.

Supplementary figure 11

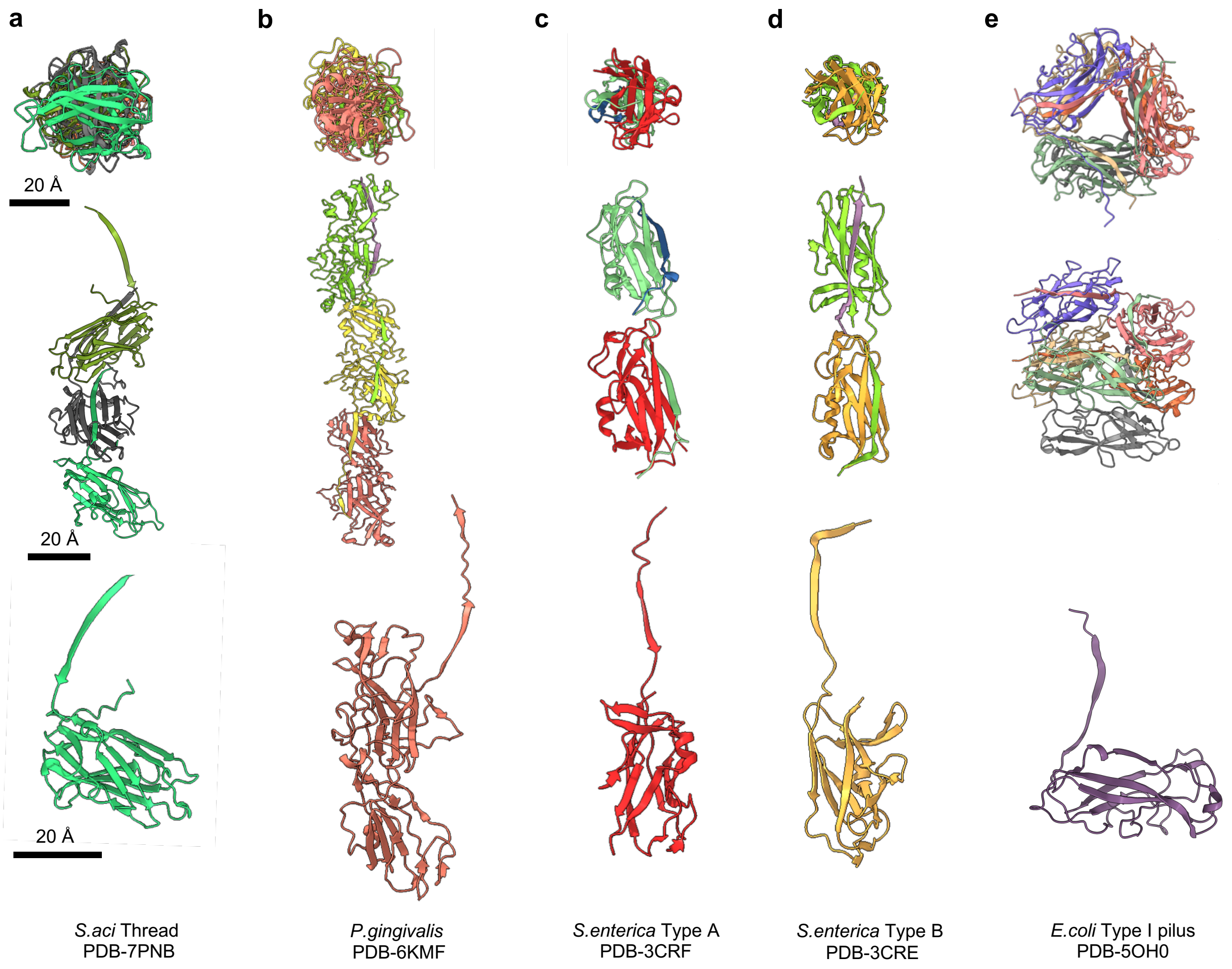

Supplementary figure 11 – Comparison between threads and bacterial pili with donor strand complementation

a-e, The structures of the *S. acidocaldarius* thread, PDB accession code 7PNB (**a**), *P. gingivalis* T5P, PDB accession code 6KMF (**b**), *S. enterica* Type A, PDB accession code 3CRF (**c**), Type B Saf pilus, PDB accession code 3CRE (**d**) and *E.coli* T1P, PDB accession code 5OH0 (**e**). Top panel, filament cross section; middle panel, filament side views; bottom panel, subunit structure for each filament. Scale bars, 20 Å

Supplementary figure 12

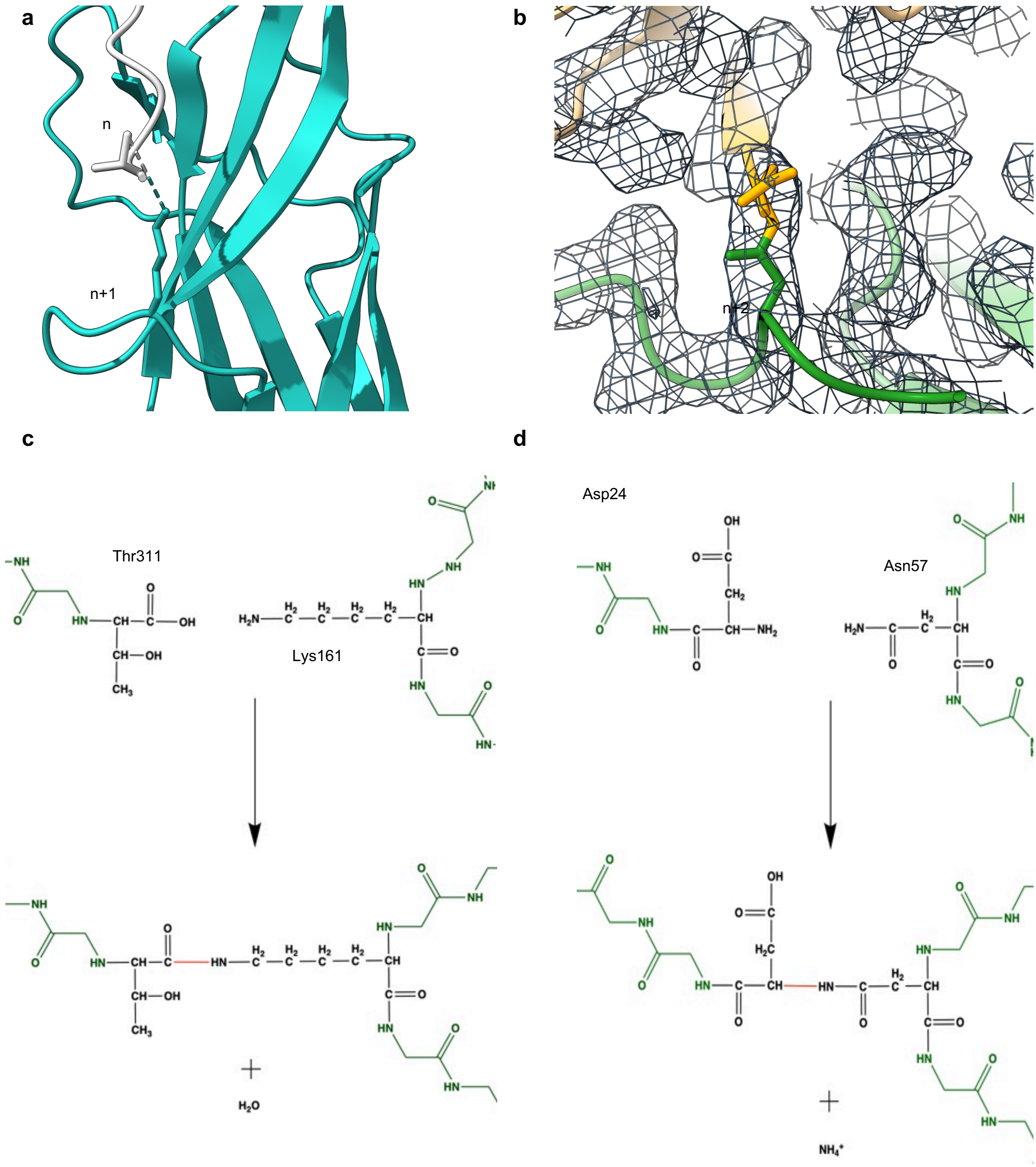

Supplementary Figure 12 – Isopeptide bond comparison between Gram positive pili and archaeal pili. a, Isopeptide bond between two adjacent Spy0128 proteins of *S. pyogenes,* where subunit n is seen in white and n-1 in cyan. **b,** Isopeptide bond between subunit n (orange) and n-2 (green) as seen in the thread filament of *S. acidocaldarius.* The chemical reaction for isopeptide bond formation in Spy0128 **(c)** *S. acidocaldarius* **(d)**, where the green residues represent the backbone, and the red line highlights the formed isopeptide bond.

Supplementary figure 13

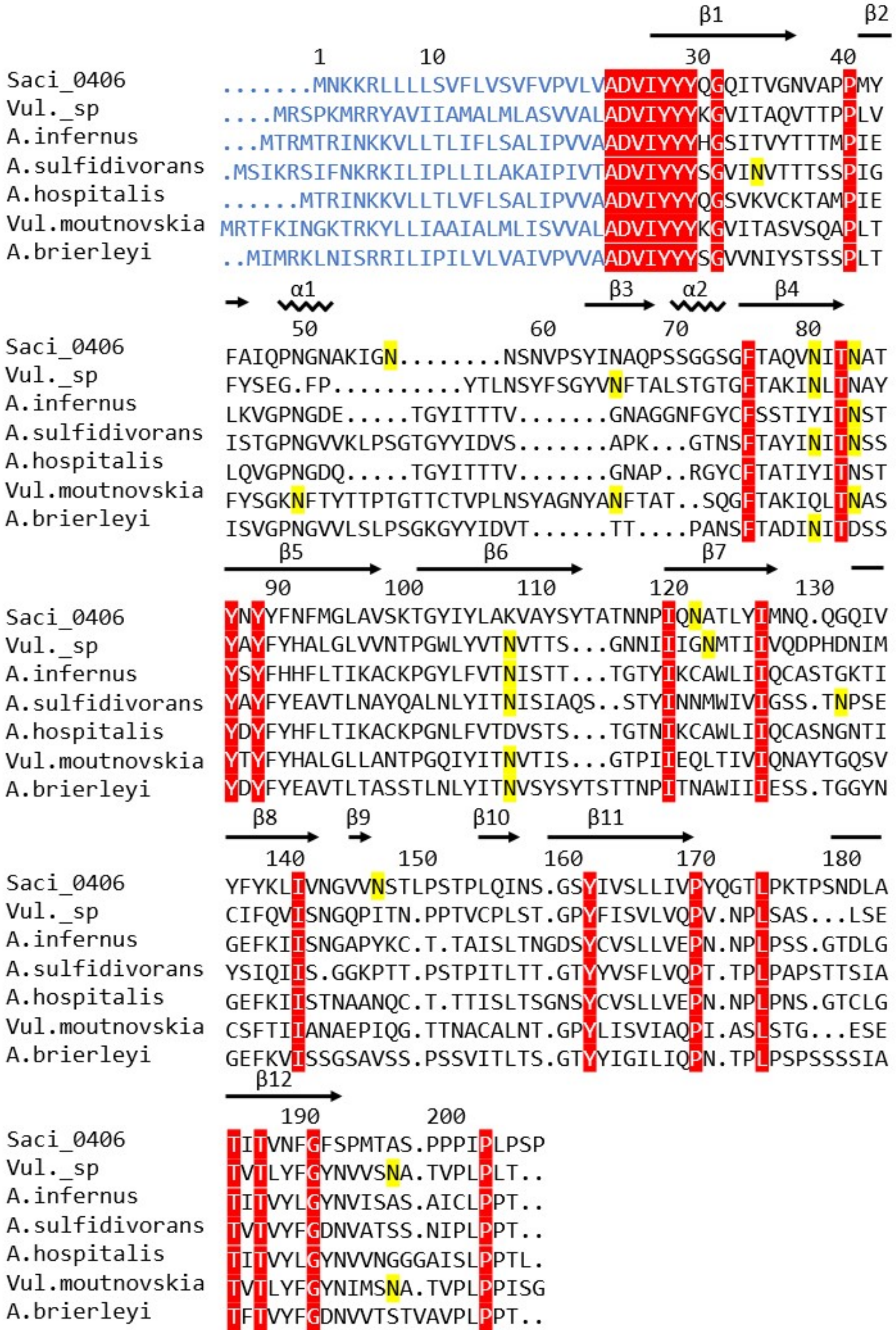

Supplementary Figure 13 – Multisequence alignment between Saci_0406 homologs in related archaeal species. Residues highlighted in red represent amino acids conserved in all species investigated. N residues highlighted in yellow represent N-glycosylation motifs (NXS/T).

Supplementary figure 14

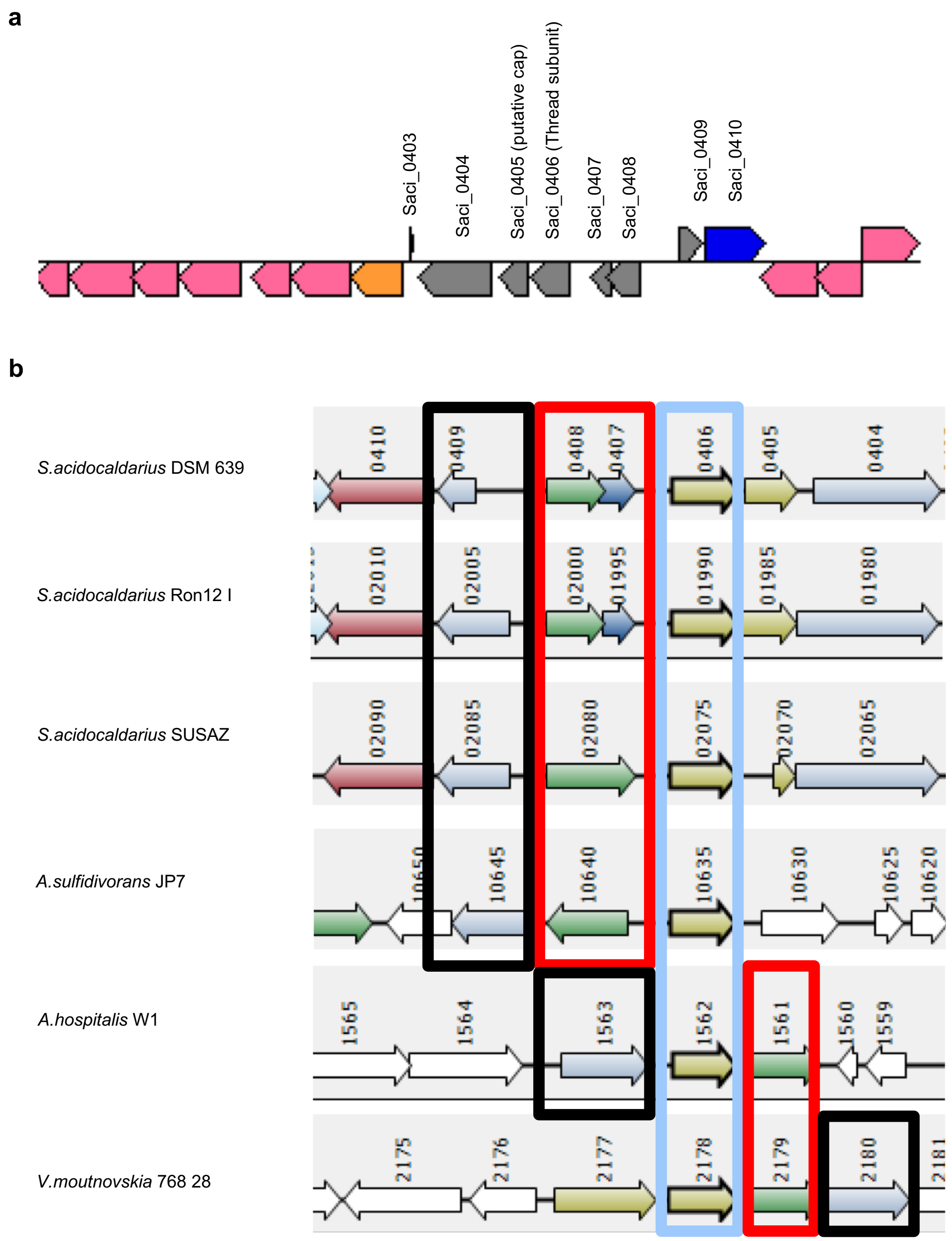

Supplementary Figure 14 – The thread gene cluster

a, gene cluster surrounding *saci_0406*. *saci_0406* encodes for the thread monomer. *saci_0405* encodes for a hypothetical cap protein. *saci_0407* and *saci_0408* may form parts of the assembly machinery. **b,** diagram visualising the similarities between genes in different strains of *S. acidocaldarius* and other related species. Red box, homologues of *saci_0407* and *saci_0408*. In the *S. acidocaldarius* strains DSM 639 and Ron12, *saci_0407* and *saci_0408* are two separate genes. In the *S. acidocaldarius* SUSAZ strain, the *saci_0407* and *saci_0408* homologs are fused into one gene, SUSAZ_02080. Blue box, homologs of the thread subunit gene (*saci_0406*). Black box, *saci_0409* homologues in the various strains and species. The sequence length of *saci_0409* seems to be variable between different organisms, but the gene is always present.

Supplementary figure 15

a

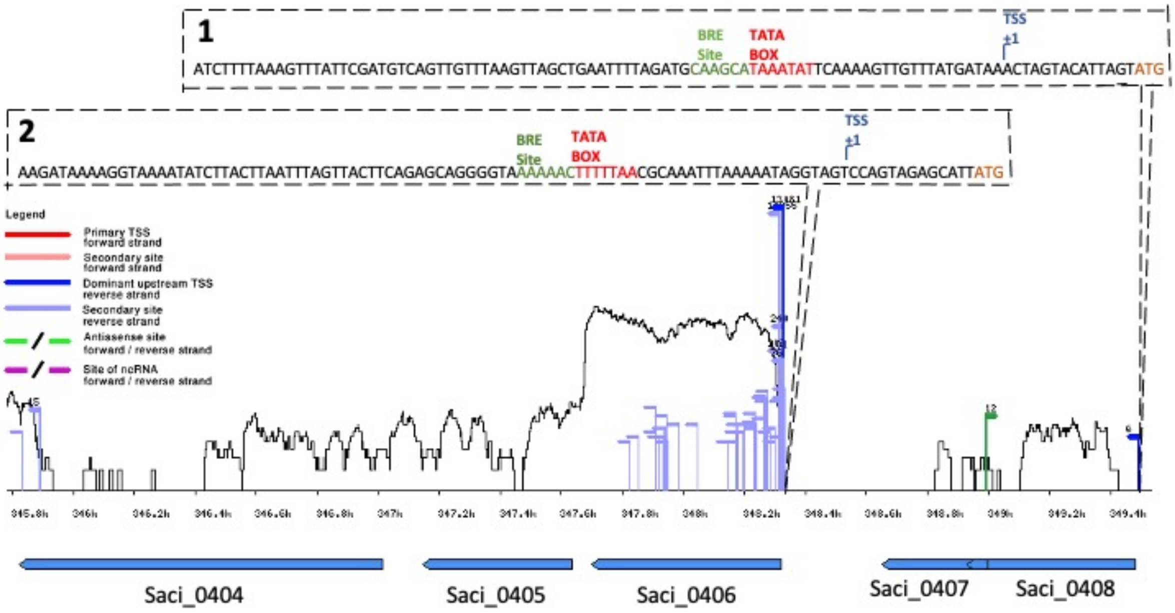

b

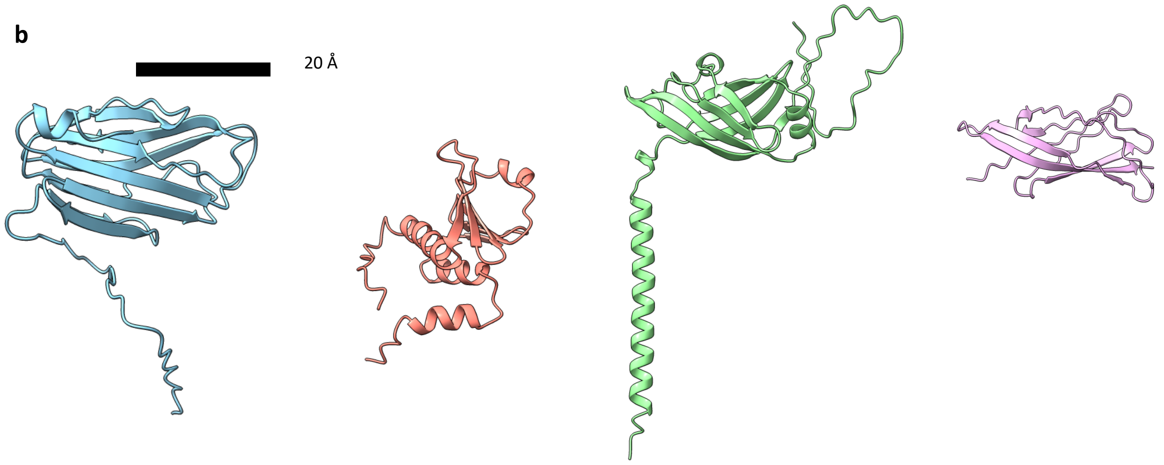

Saci_0405 Saci_0407 Saci_0408 Saci_0409

Supplementary Figure 15 – Transcriptomics profile and Alphafold2 predictions for the gene products of the proposed thread operon

a, transcriptomics profile four the proposed thread gene cluster. the transcription start sites, as well as TATA boxes and BRE sites are shown for the genes: **1,** *saci_0408*, and **2,** *saci_0406*. **b,** gallery of structural predictions for the proteins encoded in the proposed gene cluster surrounding *saci_0406*. Structures were predicted using Alphafold2[3]. Scale bar 20 Å

Supplementary figure 16

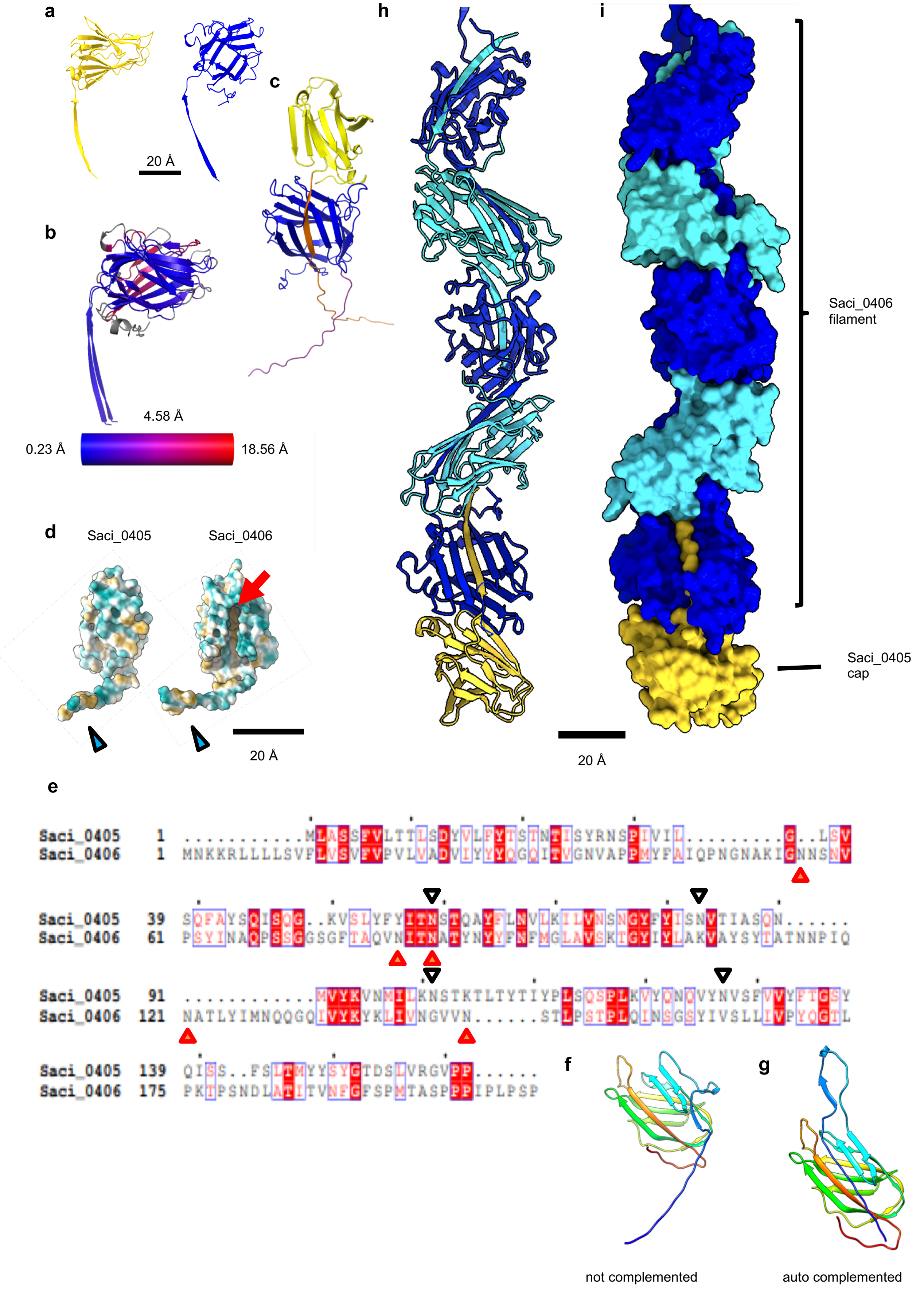

Supplementary Figure 16 – The putative cap protein Saci_0405

a, Alphafold2[3] prediction of Saci_0405 (yellow) compared with the experimentally determined structure of the thread subunit Saci_0406 (blue). **b**, alignment between the Saci_0405 prediction and the structure of Saci_0406 with RMSD value colored from blue (lowest) to red (highest), highlighting the structural similarity between both proteins. The average RMSD is 4.58 Å. **c,** Alphafold2[3] model of the Saci_0406 (blue) and Saci_0405 (yellow) complex predicts DSC (donor strand in orange). **d**, Saci_0405 and Saci_0406 shown in solid representation and coloured by hydrophobicity (orange hydrophobic, blue, hydrophilic). Both proteins have conserved tail domains that can act as beta-strand donors (blue arrowheads). However, Saci_0405 lacks the acceptor groove for a tail domain of a subsequent monomer, which is present in Saci_0406 (red arrow). This suggests that Saci_0405 could act as a cap protein. **e**, sequence alignment between Saci_0405 and Saci_0406. Black and white triangles show the glycosylation sites for Saci_0405, red and orange triangles show the glycosylation sites for Saci_0406. **f, g,** Alphafold predictions occasionally show solutions where the N-terminus of Saci_0406 complements the acceptor grove within the same molecule (auto complementation). **h**, **i**, proposed filament structure of Saci_0406 monomers (blue) with Saci_0405 modelled as the terminal cap in ribbon (h) and surface representation (i). Glycans have been omitted for simplicity. Scale bar 20 Å

Supplementary figure 17

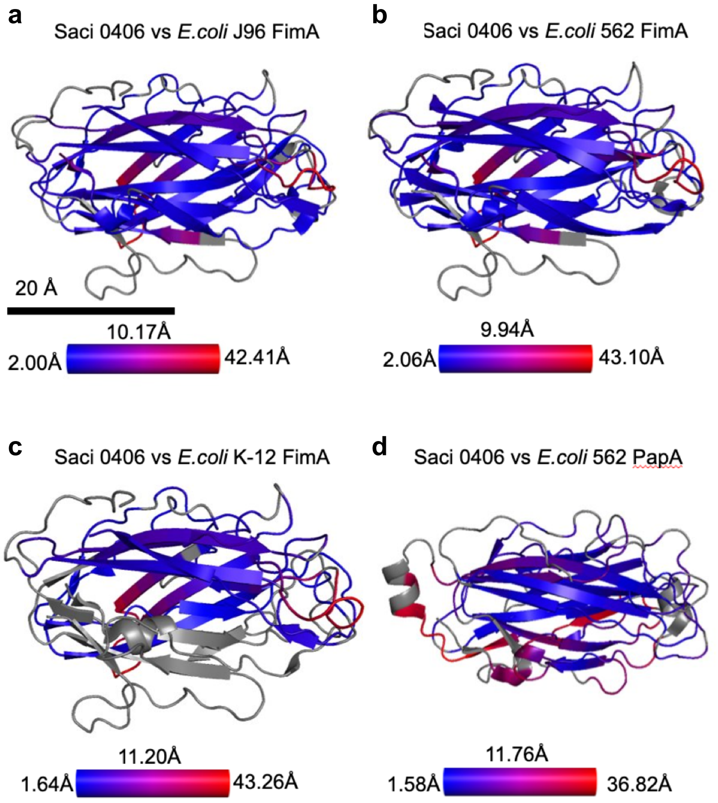

e

```
S.acidocaldarius_Saci_0406      MNKKRLLLLSVFLVSVFVPVLVADVIYYYQGQIT--------------------------    34
E.coli_562_papA                 ----------------------APTIPQGQGKVTFNGTVVDAPCSISQKSADQSIDFGQL    38
E.coli_J96_fimA                 -----------------------AATTVNGGTVHFKGEVVNAACAVDAGSVDQTVQLGQV    37
E.coli_562_fimA                 MKIKTLAIVVLSALSLSSTAALAAATTVNGGTVHFKGEVVNAACAVDAGSVDQTVQLGQV    60
E.coli_K-12_fimA                -----------------------AATTVNGGTVHFKGEVVNAACAVDAGSVDQTVQLGQV    37
                                                        .     * :

S.acidocaldarius_Saci_0406      -------VGNVAPPMYFAIQPNGNAKIGNNSNVPSYINAQPSSGGSGFTAQVNITNATYN    87
E.coli_562_papA                 SKSFLE-AGGVSKPMDLDIELVNCDIT------------AFKGGNGAKK---------GT    76
E.coli_J96_fimA                 RTASLAQEGATSSAVGFNIQLNDCDTN------------VASKA----------------    69
E.coli_562_fimA                 RTASLAQEGATSSAVGFNIQLNDCDTN------------VASKA----------------    92
E.coli_K-12_fimA                RTASLAQEGATSSAVGFNIQLNDCDTN------------VASKA----------------    69
                                        * .:  : : *:  .                   . .

S.acidocaldarius_Saci_0406      YYFNFMGLAVSKTGYIYLAKVAYSYTATNNPIQNATLYIMNQQGQIVYKYKLIVNGVVNS    147
E.coli_562_papA                 VKLAFTGPIV-NGHSDELDTNG-------GTGTA---IVVQGAGKN-----VVFDGSE--    118
E.coli_J96_fimA                 -AVAFLGTAIDAGHTNVLALQS-SA---AGSATNVGVQILDRTGAA-----LTLDGAT--    117
E.coli_562_fimA                 -AVAFLGTAIDAGHTNVLALQS-SA---AGSATNVGVQILDRTGAA-----LTLDGAT--    140
E.coli_K-12_fimA                -AVAFLGTAIDAGHTNVLALQS-SA---AGSATNVGVQILDRTGAA-----LTLDGAT--    117
                                  . * *  :       *   .       .        :::  *       : .:*

S.acidocaldarius_Saci_0406      TLPSTPLQINSGSYIVSLLIVPYQGTLPK----------TPSNDLATITVNFGFSPMTAS    197
E.coli_562_papA                 ---GDANTLKDGEN-----VLHYTAVVKKSSAVGAAVTEGAFSAVANFNLTYQ-------    163
E.coli_J96_fimA                 --FSSETTLNNGTN-----TIPFQARYFA----TGAATPGAANADATFKVQYQ-------    159
E.coli_562_fimA                 --FSSETTLNNGTN-----TIPFQARYFA----TGAATPGAANADATFKVQYQ-------    182
E.coli_K-12_fimA                --FSSETTLNNGTN-----TIPFQARYFA----TGAATPGAANADATFKVQYQ-------    159
                                   .    ::.*        : : .                 .  *.:.: :

S.acidocaldarius_Saci_0406      PPPIPLPSP      206
E.coli_562_papA                 ---------      163
E.coli_J96_fimA                 ---------      159
E.coli_562_fimA                 ---------      182
E.coli_K-12_fimA                ---------      159
```

Supplementary Figure 17 - Structural similarity between Saci_0406 and known *E. coli* chaperone-usher pilins

a – d, RMSD values calculated for pairs of Saci_0406 and *E. coli* Type-I chaperone-usher pilins, where blue indicates low values, red high, and grey areas ignored. **a,** average RMSD 10.17 Å, minimum value 2.00 Å, maximum value 42.41 Å. **b,** Average RMSD 9.94 Å, minimum value 2.06 Å, maximum value 43.10Å. **c,** average RMSD 11.20Å, minimum value 1.64 Å, maximum value 43.26 Å. **d,** Average RMSD 11.76 Å, minimum value 1.58 Å, maximum value 36.82 Å. Scale bar 20 Å**. e**, multisequence alignment of Saci_0406 and known *E. coli* type-I chaperone-usher pilins.

Supplementary References

1. Punjani, A., Rubinstein, J. L., Fleet, D. J. & Brubaker, M. A. cryoSPARC: algorithms for rapid unsupervised cryo-EM structure determination. *Nat Methods* **14**, 290–296 (2017).
2. Cramer, P. AlphaFold2 and the future of structural biology. *Nat Struct Mol Biol* **28**, 704–705 (2021).
3. Hallgren, J. *et al.* DeepTMHMM predicts alpha and beta transmembrane proteins using deep neural networks. *bioRxiv* 2022.04.08.487609 (2022) doi:10.1101/2022.04.08.487609.
