# Supplementary material for: Electron cryo-microscopy reveals the structure of the archaeal thread filament
Source: Nat Commun. 2022 Dec 1;13:7411. doi: 10.1038/s41467-022-34652-4 (PMC9715654; doi:10.1038/s41467-022-34652-4)
Supplement: Supplementary file 2 — Description of Additional Supplementary Files [file 41467_2022_34652_MOESM2_ESM.pdf]

Description of Additional Supplementary Files:

Supplementary Movie 1: Closeup of the proposed isopeptide bond (dashed line) between the N-terminal residue Asp24 of subunit n+2 (carbons in ice blue) and Asn57 of subunit n (two positions along the thread filament; carbons in gold).
