# Supplementary material for: Electron cryo-microscopy reveals the structure of the archaeal thread filament
Source: Nat Commun. 2022 Dec 1;13:7411. doi: 10.1038/s41467-022-34652-4 (PMC9715654; doi:10.1038/s41467-022-34652-4)
Supplement: Supplementary file 3 — Reporting Summary [file 41467_2022_34652_MOESM3_ESM.pdf]

nature portfolio

Corresponding author(s): Bertram Daum

Last updated by author(s): Oct 25, 2022

Reporting Summary

Nature Portfolio wishes to improve the reproducibility of the work that we publish. This form provides structure for consistency and transparency in reporting. For further information on Nature Portfolio policies, see our Editorial Policies and the Editorial Policy Checklist.

Statistics

For all statistical analyses, confirm that the following items are present in the figure legend, table legend, main text, or Methods section.

n/a	Confirmed	
☒	☐	The exact sample size (n) for each experimental group/condition, given as a discrete number and unit of measurement
☒	☐	A statement on whether measurements were taken from distinct samples or whether the same sample was measured repeatedly
☒	☐	The statistical test(s) used AND whether they are one- or two-sided<br>*Only common tests should be described solely by name; describe more complex techniques in the Methods section.*
☒	☐	A description of all covariates tested
☒	☐	A description of any assumptions or corrections, such as tests of normality and adjustment for multiple comparisons
☒	☐	A full description of the statistical parameters including central tendency (e.g. means) or other basic estimates (e.g. regression coefficient) AND variation (e.g. standard deviation) or associated estimates of uncertainty (e.g. confidence intervals)
☒	☐	For null hypothesis testing, the test statistic (e.g. F, t, r) with confidence intervals, effect sizes, degrees of freedom and P value noted<br>*Give P values as exact values whenever suitable.*
☒	☐	For Bayesian analysis, information on the choice of priors and Markov chain Monte Carlo settings
☒	☐	For hierarchical and complex designs, identification of the appropriate level for tests and full reporting of outcomes
☒	☐	Estimates of effect sizes (e.g. Cohen's d, Pearson's r), indicating how they were calculated

Our web collection on statistics for biologists contains articles on many of the points above.

Software and code

Policy information about availability of computer code

Data collection	Data were collected using the EPU 1.10.0.65 software, running on a Titan Krios microscope.
Data analysis	For data anlysis CryoSPARC 3.2.0 and 3.3.0, EMAN2-2016.2, Relion3.1, Chimera1.15, ChimeraX1.4, DeepEMhancer0.11, SignalP5, PSI-BLAST, REFMAC5.8.0267, CCPEM1.5.0, AlphaFold2, ColabFold, PDBefold, Clustal Omega, MOLREP11.7.03, Phenix1.19.2-4158, and Coot0.9.6, JLigand 2.6 and CCP4 7.1 were used.

For manuscripts utilizing custom algorithms or software that are central to the research but not yet described in published literature, software must be made available to editors and reviewers. We strongly encourage code deposition in a community repository (e.g. GitHub). See the Nature Portfolio guidelines for submitting code & software for further information.

Data

Policy information about availability of data

All manuscripts must include a data availability statement. This statement should provide the following information, where applicable:

- Accession codes, unique identifiers, or web links for publicly available datasets
- A description of any restrictions on data availability
- For clinical datasets or third party data, please ensure that the statement adheres to our policy

The atomic coordinates and electron density map were deposited in the Protein Data Bank (https://www.rcsb.org/) with accession number 7PNB (DOI: 10.2210/pdb7pnb/pdb) and in the EM DataResource (https://www.emdataresource.org/) with the accession number EMDB–13546 (https://www.emdataresource.org/EMD-13546). The S. acidocaldarius genome was analysed using the KEGG database (https://www.genome.jp/kegg/). The genome of S. acidocaldarius (strain

DSM639), can be accessed via the KEGG accession code T00251 (https://www.genome.jp/entry/gn:T00251) or the NCBI gene bank code CP000077 (https://www.ncbi.nlm.nih.gov/nuccore/CP000077). The transcriptomics data analysed in this study can be accessed in the Pan Genomic Database for Genomic Elements Toxic To Bacteria under the following link:
https://exploration.weizmann.ac.il/TCOL/index_singleOrg.php?organism=sulfolobus_acidocaldarius&tab=0.

Human research participants

Policy information about studies involving human research participants and Sex and Gender in Research.

Reporting on sex and gender	N/A
Population characteristics	N/A
Recruitment	N/A
Ethics oversight	N/A

Note that full information on the approval of the study protocol must also be provided in the manuscript.

Field-specific reporting

Please select the one below that is the best fit for your research. If you are not sure, read the appropriate sections before making your selection.

- [x] Life sciences
- [ ] Behavioural & social sciences
- [ ] Ecological, evolutionary & environmental sciences

For a reference copy of the document with all sections, see nature.com/documents/nr-reporting-summary-flat.pdf

Life sciences study design

All studies must disclose on these points even when the disclosure is negative.

Sample size	The number of particles used in the final structure was determined from the original particles extracted from micro-graphs. Then 2D classification jobs followed by 3D refinements were carried out, giving a final total of 188,620 particles in the final reconstruction.
Data exclusions	No data was excluded
Replication	CryoEM structures are averages of hundred's of thousands of protein particles. The resulting 3D maps undergo a rigorous validation process, complying to standards that are widely accepted in the field. The image processing pipeline is iterative, meaning that 3D maps are reproduced several times. There were no instances at which the cryoEM map could not reproduced during this process, as the data continuously improved in resolution.
Randomization	Randomization was not relevant to this study, as statistical analyses were not performed.
Blinding	Blinding was not relevant to this study, as statistical analyses were not performed.

Reporting for specific materials, systems and methods

We require information from authors about some types of materials, experimental systems and methods used in many studies. Here, indicate whether each material, system or method listed is relevant to your study. If you are not sure if a list item applies to your research, read the appropriate section before selecting a response.

Materials & experimental systems

n/a	Involved in the study	
☒	☐	Antibodies
☒	☐	Eukaryotic cell lines
☒	☐	Palaeontology and archaeology
☒	☐	Animals and other organisms
☒	☐	Clinical data
☒	☐	Dual use research of concern

Methods

n/a	Involved in the study	
☒	☐	ChIP-seq
☒	☐	Flow cytometry
☒	☐	MRI-based neuroimaging
